# Innovative Approaches to Evaluate Sugar Beet Responses to Changes in Sulfate Availability

**DOI:** 10.3389/fpls.2018.00014

**Published:** 2018-01-31

**Authors:** Piergiorgio Stevanato, Chiara Broccanello, Vita M. C. Moliterni, Giuseppe Mandolino, Valeria Barone, Luigi Lucini, Giovanni Bertoldo, Marco Bertaggia, Massimo Cagnin, Diego Pizzeghello, Andrea Baglieri, Andrea Squartini, Giuseppe Concheri, Serenella Nardi

**Affiliations:** ^1^Department of Agronomy, Food, Natural Resources, Animals and Environment, University of Padova, Padova, Italy; ^2^Council for Agricultural Research and Economics, Genomics Research Centre, Fiorenzuola d’Arda, Italy; ^3^Council for Agricultural Research and Economics, Research Centre for Industrial Crops, Bologna, Italy; ^4^Department of Agriculture, Food and Environment, University of Catania, Catania, Italy; ^5^Institute of Agricultural and Environmental Chemistry, Università Cattolica del Sacro Cuore, Piacenza, Italy

**Keywords:** sugar beet yield, nutritional stress, sulfate availability, omics profiling, high-throughput qPCR profiling

## Abstract

In this study, a system based on omics profiling was set-up for sugar beet (*Beta vulgaris* L. subsp. *vulgaris*) evaluation after changes in sulfate availability. Seedlings were grown on sulfate-deprived Hoagland solution. Six days after germination, 100 μM MgSO_4_ was added to the solution. Root samples were collected 36 h after treatments. WinRHIZO root-scanning approach was used for the automated image analysis of plant root morphology. Inductively Coupled Plasma Spectrometry (ICP-OES) and quadrupole-time-of-flight mass spectrometry (Q-TOF) were used for ionomic and metabolic analysis, respectively. Nanofluidic real-time PCR (OpenArray system) was used for molecular profiling. OpenArray chips were designed with TaqMan probes for 53 sugar beet genes putatively involved in sulfate nutrition. At morphological level treated seedlings showed significantly higher values (*P* < 0.01) than untreated plants for root traits related to soil exploration and nutrient uptake, such as total root length, fine roots length and root tips number. ICP-OES, Q-TOF and transcriptomic data revealed changes due to sulfate availability in sugar beet samples. Two key results are highlighted in sulfate-supplied roots and leaves. Firstly, high expression levels of auxin efflux carrier component 1 (PIN) and 5-phosphoribosyl-anthranilate, precursor of tryptophan and auxin synthesis, were observed in roots. Secondly, high levels of 2-Cys peroxiredoxin BAS1, chloroplastic, thioredoxin reductase (NADPH) and cysteine synthase, chloroplastic/chromoplastic, *O-*acetylserine sulfhydrylase, involved in protection against oxidative stress and cysteine synthase activity, respectively, were observed in leaves. Based on our findings, the combination of evaluated omics approaches could become a key system for the evaluation of the nutritional status of sugar beet under different nutrient availability conditions.

## Introduction

Sugar beet (*Beta vulgaris* L. subsp. *vulgaris*) is an important crop that satisfies 25% of world sugar demand with a total production of 269 Mt (FAO, 2013). In the Mediterranean area, global climate change has led to a decrease of about 1 t ha^-1^ in sugar production due to water shortage and low nutrient availability (Barbanti et al., 2007). Particularly, in modern day agriculture, sulfur deficiency has become a major constraint, since its availability in the soil is gradually decreasing (Lewandowska and Sirko, 2008).

In sugar beet, sulfur is essential for protein synthesis and to keep the presence of amino and sulfur-containing compounds balanced (Hoffmann et al., 2004). S-demand and S-removal of sugar beet is 30 and 5 kg ha^-1^, respectively (Haneklaus et al., 1998). Critical leaf concentration for S-deficiency, which results in yield depression of 5%, was estimated as 3 mg g^-1^ S (d.w.) by Haneklaus et al. (2007). An unbalanced proportion between N and S, due to low S concentration, results in alfa-amino N accumulation leading to a lower sugar beet technical quality and decreasing root storage capacity (Burba, 1996). Thomas et al. (2000) reported a N/S threshold value of 20:1 in the shoot for yield reduction, whereas Haneklaus and Schnug (1996) found a lower N/S ratio of 14 as limiting value in sugar beet.

Genes related to sulfate response, able to transmit external signals and trigger adaptive changes, are well known and studied. Plants can adapt to the environment in a highly coordinated and dynamic manner (Giehl et al., 2014). This consists of multiple organization levels and links genes through different molecular pathways.

An approach to investigate the effect of changes in sulfur availability on plant composition concerns the application and integration of advanced omics technologies (Huang and Salt, 2016). The omics technologies are specifically utilized to describe the global profiling of biological matrices. Combinations of these techniques have been applied and served as high-throughput screening to allow the identification of potential specific biomarkers (Yuan et al., 2008).

Many studies attribute a fundamental role to the plant root system in the competition for survival in natural environments and the greatest selection pressure is for the acquisition of elements of soil fertility (water and nutrients), which is strictly dependent on root morpho-physiology (Gruber et al., 2013). The characterization of root phenomics is therefore essential to understand how trait variations are attributable to genotype and environmental factors (Cichy et al., 2009).

Changing metabolic homeostasis due to environmental stresses triggers the production of different proteins that could restore a new homeostasis. An integration of ionomic and metabolomic approaches could give a comprehensive assessment to understand which metabolites are involved in responses to a specific environment (Atkinson and Urwin, 2012). A specific stress response can be identified by specific metabolic fingerprinting (Shulaev et al., 2008). Moreover, target metabolite analysis combined with a dynamic gene expression profile is used to elucidate gene-to-gene and metabolite-to-gene networks through which plants coordinately modulate their responses to nutritional stresses.

In this study, a method based on the combination of different omics technologies was set-up for sugar beet (*Beta vulgaris* L. subsp. *vulgaris*) evaluation to achieve a holistic view of plant response to changes in sulfate availability. Root morphology, plant ionome and metabolome, together with gene expression profiling, were analyzed in leaves and roots of sulfate-deprived and supplied plants. In particular, we evaluated the capacity of the proposed omics techniques to depict complex plant–sulfate interactions.

## Materials and Methods

### Plant Material

The plant material used in this study was the sugar beet hybrid “Shannon” provided by Lion Seeds Co., Ltd. (Maldon, United Kingdom). It is a diploid hybrid obtained from a cross between a multigerm pollinator resistant to rhizomania (Rizor-Holly source) and a susceptible monogerm male-sterile.

### Growing Conditions

Seeds were surface-sterilized by dipping in 76% ethanol for 5 min and rinsed three times in distilled water. Seeds were germinated on distilled water-moistened filter paper in a growth chamber in the dark for 48 h at a temperature of 25°C. After germination, seedlings were transplanted to 35-liter plastic tanks containing a sulfate-deprived Hoagland solution. The tanks were placed in a growth chamber at 25/18°C and 70/90% relative humidity with a 14/10 h light/dark cycle (PPFD above shoot: 300 μE m^-2^ s^-1^) and nutrient solution was replaced daily. Six days after germination, 100 μM of MgSO_4_ was added to the solution. On the eighth day, fresh leaves and roots were harvested and stored at -80°C for further analysis.

### Ionomic Analysis

Leaf samples were digested with concentrated HNO_3_ in a microwave system. The elements concentration was determined by inductively coupled plasma ICP-OES, Ciros Vision EOP (Spectro A. I. GmbH, Germany). Elements were quantified using certified multi-element standards. Sulfate was extracted in 20 cm^3^ of Millipore water by incubation at 70°C for 30 min. The extract was centrifuged at 20,000 *g* for 30 min, and the supernatant filtered through a 0.45 μm filter unit. Sulfate content was determined by ICP-OES. This procedure was previously adopted by Stevanato et al. (2015).

### Root Morphological Analysis

Root morphological parameters (total root length, surface area and total number of tips) were determined by computerized scanning (STD 1600, Regent Instruments, Quebec, QC, Canada) and analyzed using WinRHIZO software (Regent Instruments).

### Metabolomic Analysis

An untargeted screening was conducted as previously set up (Rouphael et al., 2016). Briefly, compounds were comminuted using Ultra-Turrax and extracted in 70% methanol (added with 1% HCOOH), then analyzed using a quadrupole-time-of-flight mass spectrometer coupled to an UHPLC chromatographic system (UHPLC/Q-TOF). A 1290 UHPLC system was used coupled to a 6550 quadrupole-time-of-flight mass spectrometer and equipped with a Jet Stream ESI ionization system (all from Agilent Technologies, Palo Alto, CA, United States). Reverse-phase chromatographic separation was achieved using a Knauer BlueOrchid C18 column (100 mm × 2 mm i.d., 1.8 μm) and a mixture of water (proteomic grade, VWR, Milan, Italy) and methanol (LCMS grade, VWR, Milan, Italy) as mobile phase. Acquisition was performed in positive SCAN mode (100–1200 m/z) and compounds were then identified using accurate mass and isotopic pattern (isotopic spacing and isotopic ratio) against the database exported from PlantCyc ^1^. Metabolomic data were interpreted using Agilent Mass Profiler Professional B.12.06. Compounds were filtered by abundance and frequency (area of >5000 counts and detection in 100% of samples in at least one condition, respectively), normalized at the 75th percentile and baselined to the median of each compound in all samples. Unsupervised hierarchical cluster analysis was then conducted using the fold-change heat-map and setting the similarity measure as Euclidean and Wards as linkage rule. Partial least squares discriminant analysis (PLS-DA, N-fold validation with *N* = 4) was also performed, and variables loadings, used to build the class prediction model, plotted according to their weight within the latent vectors. Compounds with the highest scores on the first and second latent vectors were exported from the covariance structures in the PLS-DA hyperspace. The identification of differential metabolites was finally investigated by combining analysis of variance (*P* < 0.001, Bonferroni multiple testing correction) and fold-change analysis (cut-off > 5) into Volcano Plots.

### Transcriptomics Analysis

Total RNA was extracted from 100 mg of root tissues using a EuroGold TriFastTM kit (Euroclone, Italy) following the manufacturer’s recommendations. RNA was quantified with a Qubit Fluorometer (Invitrogen, Carlsbad, CA, United States) using a Qubit RNA HS Assay Kit. One microgram of total RNA was reverse transcribed using the FastGene 55-Scriptase (Nippon Genetics, Japan) in a total volume of 20 μl following the manufacturer’s recommendations. The cDNAs were used to analyze the expression level of 53 genes related to nutritional status in sugar beet (Barone et al., 2017). Real-time PCR experiments were conducted in a final volume of 5 μl containing 2.5 μl of 2× TaqMan Open Array master mix (Life Technologies, United States), and 2.5 μl of cDNA. Real-time PCR was performed on the QuantStudio 12K Flex Real-Time PCR System (Life Technologies, United States) using the following thermocycler program: 10 min pre-incubation at 95°C, followed by 50 cycles of 15 s at 95°C and 1 min at 60°C. The sequences of the primers and TaqMan probes designed for the Real-time PCR experiments are reported as Supplementary Material S1.

The comparative *C*t method was used to analyze the genes relative expression (Livak and Schmittgen, 2001). Data were normalized against the average transcript abundance of three housekeeping genes (*Tubulin, Bv2_037220_rayf; GAPDH, Bv5_107870_ygnn; Histone H3, Bv6_127000_pera*). The fold change in expression of genes was calculated using the formula 2^-ΔΔCt^, where ΔΔC_t_ = (C_t_ target gene - average C_t_ reference genes)_treatment_- (C_t_ target gene - average C_t_ reference genes)_control_. All data are the means of three biological replicates, each one composed of three technical replicates ±SE of one representative experiment. The *C*_t_ method was used to quantify the relative gene expression levels and the results expressed as 2^-ΔCt^, where ΔC_t_ = (C_t_ of reference gene - C_t_ of target gene).

### Data Analysis

A completely randomized experimental design was adopted with five replications per treatment and 60 seedlings per replicate. All data were subjected to the normality test (Kolmogorov–Smirnov) and homogeneity of variance (Levene-Median). A factorial ANOVA was conducted using the statistical software package Statistica v. 13.0 (Dell Inc., United States) to investigate the effect of different treatments, tissues, genes and their interactions.

## Results

**FIGURE 1:**
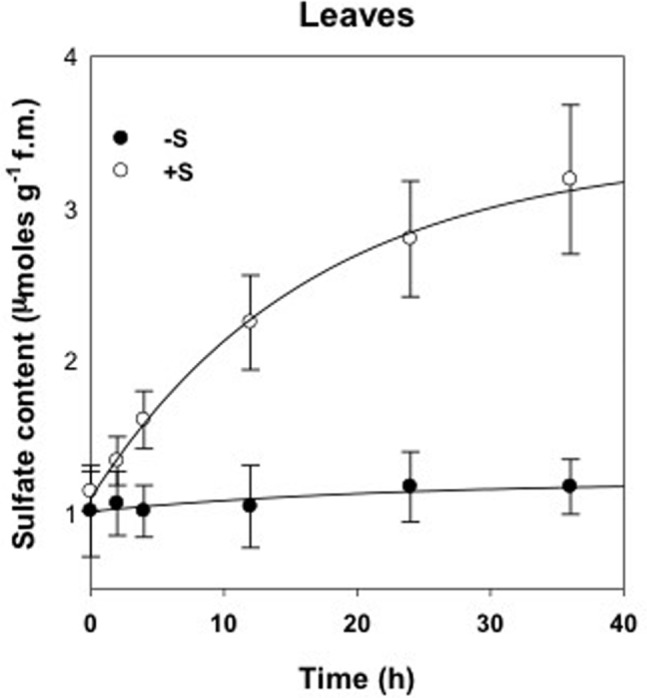
Sulfate content in leaves of deprived control plants (-S) and supplied with 100 μM of MgSO_4_ for 36 h (+S).

The accumulation dynamics of sulfate was evaluated for a period of 36 h in deprived control plants (-S) and supplied plants (+S) (**Figure 1**). Sulfate content increased strongly in leaves of treated plants and, as expected, remained mostly stable in deprived plants. After 36 h of treatment with 100 μM of MgSO_4_, the sulfate contents of leaves were significantly higher (*P* < 0.01) than those of the deprived plants.

**FIGURE 2:**
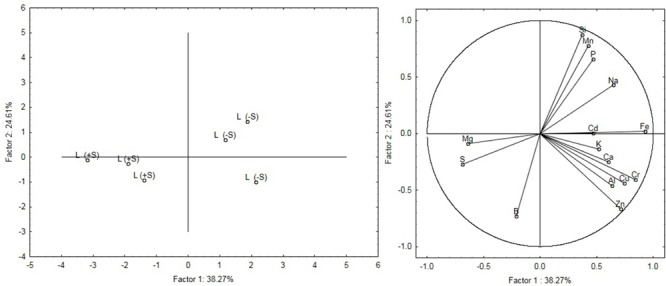
Principal components analysis (PCA) of the leaves ionome. The figure on the Left shows the modification of the leaves ionome as a function of the nutritional regime (S-deficiency and S-sufficiency). The figure on the Right shows the relationship between variables and principal components and also highlights relationships between the variables themselves.

Leaves were analyzed with ICP-OES in order to reveal the effect of changes in sulfate availability on the ionome profile (**Table 1**). No significant increases were detected in the elements concentration in response to S nutrition, except for S and Mg. S concentration significantly increased (*P* < 0.01) in leaves (+162%) in treated compared to untreated plants. A significant difference (*P* < 0.05) in Mg concentration was observed in leaves of treated plants, which showed a 4.5% increase. Samples within treatments showed a clear separation as detected by the principal component analysis (PCA) (**Figure 2**). Factors 1 and 2 explained 38.27% and 24.61% of the total variation, respectively. Factor 1 is related to the variation of S and Mg in leaves ionome.

**Table 1:** Leaf concentration of mineral elements of deprived control plants (-S) and supplied plants with 100 μM of MgSO_4_ for 36 h (+S).

Samples Treatment	Leaf	
	+S	-S
**Elements**		
Al	9.6 ± 1.6	7.7 ± 3.0
B	17.3 ± 1.0	16.1 ± 0.9
Ba	6.6 ± 1.0	6.5 ± 0.7
Ca	2929.8 ± 534.3	3375.7 ± 207.8
Cd	0.3 ± 0.0	0.3 ± 0.0
Cr	0.4 ± 0.1	0.4 ± 0.1
Cu	12.9 ± 0.8	16.7 ± 4.4
Fe	152.7 ± 7.3	137.7 ± 10.4
K	75147.6 ± 5225.3	72134.2 ± 1699.0
Mg	4969.2 ± 532.0	4086.2 ± 169.1^*^
Mn	91.8 ± 7.9	88.0 ± 3.6
Na	841.1 ± 84.9	781.2 ± 73.9
P	9153.2 ± 635.2	10387.9 ± 382.2
S	2599.7 ± 261.3	994.0 ± 40.3^*^*
Si	20.7 ± 1.6	16.9 ± 1.3
Zn	42.7 ± 6.8	45.9 ± 4.2

*The concentration of elements (mg kg^-*1*^ DW) is expressed as mean ±SE of three biological replicates and each replicate consisted of sixty seedlings. Significant difference between treatments was determined by ANOVA and marked as ^∗^*P* < 0.05 and ^∗∗^*P* < 0.01.*

**FIGURE 3:**
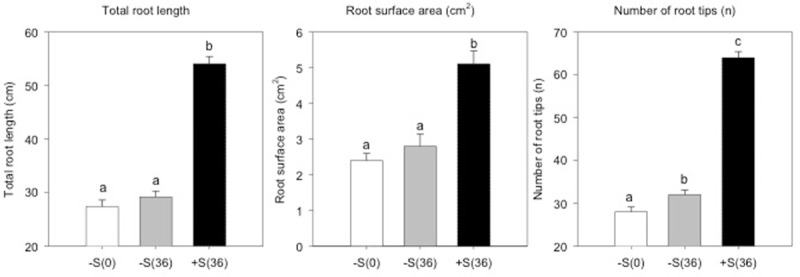
Total root length, root surface area (cm^2^) and number of root tips of sulfur deprived plants (–S(0)), sulfur deprived plants after 36 h (–S(36)) and sulfur supplied plants after 36 h (+S(36)).

To evaluate the sulfate treatment effects on root apparatus, we studied three different parameters: total root length, surface area and number of tips. As represented in **Figure 3**, plants grown in 100 μM of sulfate solution show significantly higher values (*P* < 0.01) for all the parameters analyzed than deprived plants. In addition, the data obtained reveal that the mechanism actuated by deprived plants, in response to an additional 36 h of sulfate deprivation, is expressed in a significant increase (*P* < 0.01) in the number of root tips.

**FIGURE 4:**
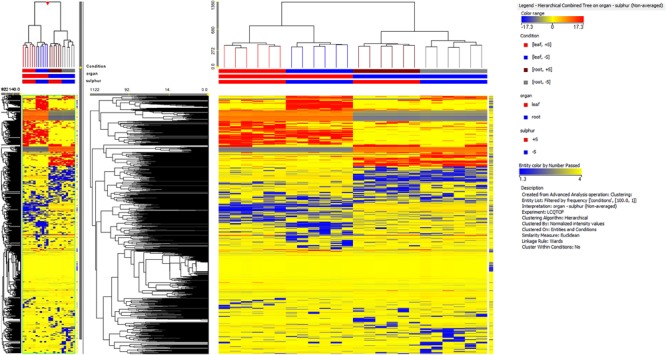
Not averaged unsupervised cluster analysis in sugar beet roots and leaves of supplied and deprived plants (similarity: Euclidean; linkage rule: Ward). Compound intensity was used to build up heat maps, on the basis of which the clusters were generated.

**FIGURE 5:**
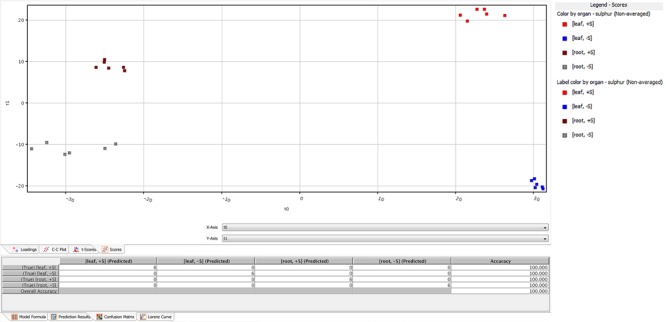
Partial least squares discriminant analysis (PLS-DA) conducted from the UHPLC-/QTOF metabolite profiling in sugar beet roots and leaves of supplied and deprived plants. Samples distribution in the hyperspace of the class prediction model is provided in the Upper, while the compounds loading plot is provided in the Lower one.

A total of 2,400 metabolites were identified via UHPLC/Q-TOF. The unsupervised cluster analysis was performed on the dataset to better focus on the effect of the sulfate treatment (**Figure 4**). The results showed that the two treatments were properly grouped in both roots and leaves, thus indicating the presence of a chemical signature from the treatment in the metabolomics dataset. The output of PLS-DA, shown in **Figure 5**, consistently indicated a good discrimination between treatments on the basis of their metabolic profile. Indeed, the class prediction model gave good accuracies for both tissues and treatments (overall accuracy of 100%). The compounds selected from the Volcano analysis of plants treated with 100 μM of MgSO_4_ for 36 h (using a fold change cut off >5 and a *p-*value of 0.001) are reported as Supplementary Material S2. Most of the differential compounds identified were free amino acids such as tryptophan, proline, lysine, glutamate, glutamine and cysteine. The Volcano analysis also revealed high levels of *O*-acetyl-*L*-serine, Jasmonic acid and 12-hydroxy-jasmonoyl-L-isoleucine in leaves.

**FIGURE 6:**
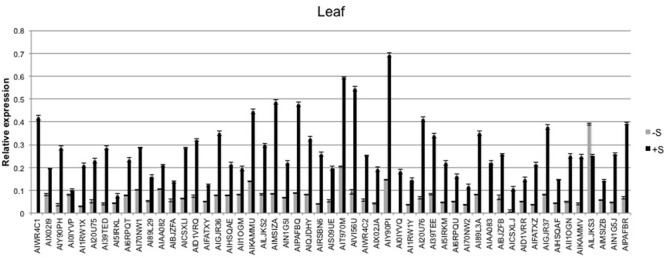
Average relative expression of the 53 sulfur related gene in deprived and supplied leaves. The *C*_t_ method was used to quantify the relative gene expression levels and the results expressed as 2^-Δ^*^C^*^t^, where ΔC_t_ = (C_t_ of reference gene - C_t_ of target gene).

**FIGURE 7:**
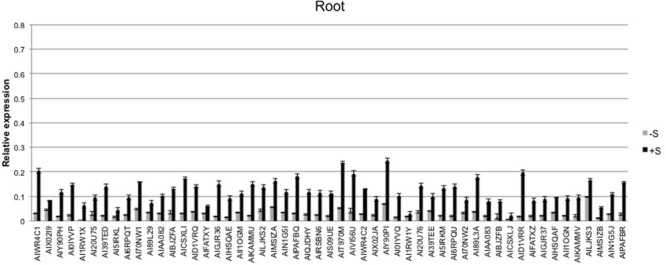
Average relative expression of the 53 sulfur related genes in deprived and supplied roots. The *C*_t_ method was used to quantify the relative gene expression levels and the results expressed as 2^-Δ^*^C^*^t^, where ΔC_t_ = (C_t_ of reference gene - C_t_ of target gene).

The expression level of the 53 sulfate-related genes was evaluated in deprived and supplied leaves and roots. The ANOVA showed a significant effect of treatments (*P* < 0.01) and tissues (*P* < 0.01), as well as genes (*P* < 0.01) (**Table 2**). Relative expression of 53 genes in leaves (**Figure 6**) was significantly higher compared to roots (**Figure 7**) of treated and untreated plants. Moreover, gene expression analysis revealed that the highest relative quantity is shown in supplied roots by Flavonol sulfotransferase-like (AIY90PI, Bv6_137840_uaap), 28 kDa ribonucleoprotein, chloroplastic (AIT970M, Bv7u_180460_dcmt) and Glutamate/leucine/phenylalanine/valine dehydrogenases (AIWR4C1, Bv3_057000_nenr). A sixfold increase of the gene auxin efflux carrier component 1 (AI1RW1X, Bv3_065290_srwc) was detected in roots compared to leaves. Supplied leaves have a high relative quantity of the same three genes highly expressed in roots. In particular, Flavonol sulfotransferase-like showed a onefold increase compared to roots. However, supplied leaves are subjected to a significant expression of other genes: Aspartic proteinase-like protein 1 (AIVI56U, Bv_24910_jato), 3-ketoacyl-CoA synthase 17 (AIMSIZA, Bv7_156890_eowm) and cysteine synthase, chloro plastic/chromoplastic, O-acetylserine sulfhydrylase (AIGJR36, Bv1_004580_xnrs). Gene coding for thioredoxin reductase (NADPH) (AIKAMMV, Bv3_063630_mpup) and 2-Cys peroxiredoxin BAS1, chloroplastic (AII1OGN, Bv7_157460_rcod) were twofold and onefold more expressed in leaves than roots, respectively.

**Table 2:** Analysis of variance (ANOVA) showing the effect of different treatments, tissues and genes (^∗^*P* < 0.05; ^∗∗^*P* < 0.01, factorial ANOVA test) on the expression of 53 sugar beet genes putatively involved in sulfate nutrition.

Effect	*df*	*SS*	*MS*	*F*	*P*
Treatment	1	1690	1690	152.9	^∗∗^
Tissue	1	9431	9431	853.2	^∗∗^
Gene	52	3124	57	5.1	^∗^

## Discussion

Many studies have focused their attention on explaining plant nutritional stress using an integrated omics approach (Saito and Matsuda, 2010). In particular, sulfur starvation has been extensively studied for the model plant *Arabidopsis* (Nikiforova et al., 2005, 2006). In this work, sulfur shortage, one of the main sugar beet nutritional deficiencies that causes significant sugar yield losses, is dissected by means of omics technologies to set up a method able to detect and describe this nutritional limitation. The 53 genes used in this study were selected on the basis of a previous experiment of RNA-seq analysis and validated for their involvement in plant responses to nutritional changes (Barone et al., 2017). The product of these genes plays important roles in many biological processes, such as biosynthesis, response to stress, cellular amino acid metabolism, transport, and sulfur compound metabolism. Changes in root morphology, plant ionome, metabolome and gene expression profile, in leaves and roots of sulfate-deprived and supplied sugar beet plants, highlighted the presence of potential biomarkers involved in sulfate nutrition. These biomarkers are mainly related to auxin synthesis, plant protection, amino acid and cysteine biosynthesis.

Root morphology is closely related to plant S uptake efficiency, especially the root length, surface area and number of tips (Stevanato et al., 2015). These parameters show a significant difference in plants treated with 100 μM of MgSO_4_ for 36 h compared to sulfur deprived plants. This has also been observed in the soil, since sulfur is mobile and present in the deeper soil profile and stimulates plants to rapidly elongate the primary root (Zhao et al., 2014). From a transcriptomics and metabolomics point of view, this is reflected in a high expression of auxin efflux carrier component 1 (PIN) and in the up regulation of 5-phosphoribosyl-anthranilate, precursor of tryptophan and auxin synthesis. The root morphological analysis also revealed a significant increase in the number of root tips in plants maintained at sulfur deficiency for 36 h. Root tips have a fundamental role in nutrients acquisition and the perception of nutritional stress (López-Bucio et al., 2003). A previous study on *Arabidopsis* reported that sulfate-deprived plants have a larger number of root tips and fine roots, increasing the root/shoot ratio (Gläser et al., 2014). In addition, Zhao et al. (2014) observed that sulfate deprivation stimulated cell division activity and root tip expansion.

Plants grown without sulfur show different mineral composition from plants grown with 100 μM of MgSO_4_ as highlighted by ICP-OES analysis. The leaf sulfur content of the treated plants was much lower than the threshold value below which significant production losses were observed by Haneklaus et al. (2007). PCA analysis of mineral elements revealed that leaves and root samples belonged to separate clusters corresponding to their nutritional regime (S-deficiency and S-sufficiency). Plants treated with sulfur show higher Mg levels than S-deprived ones, demonstrating that these nutrients are correlated with each other (Dietz, 1989). Sulfur is essential for chlorophyll formation and magnesium is the central core of the chlorophyll molecule. In sugar beet, a decrease of chlorophyll under sulfur deficiency has already been described by Thomas et al. (2000).

Many genes highly expressed in the leaves supplied with 100 μM of MgSO_4_ are involved in stress response, in particular 2-Cys peroxiredoxin BAS1, chloroplastic and thioredoxin reductase (NADPH). The first one is an antioxidant enzyme that has an important role in cell protection against oxidative stress and is involved in protecting photosynthesis (Dietz et al., 2002). The second is involved in regulation of chlorophyll biosynthetic process and in the removal of superoxide radicals. Furthermore, the enhanced defense activity was also observed at metabolic levels with an up regulation of Jasmonic acid and 12-hydroxy-jasmonoyl-L-isoleucine in leaves, which have a crucial role in mediating plant stress response.

The presence of sulfur in the form of cysteine residues is essential for the conformation and stability of proteins. Transcriptomics analysis shows an over expression of cysteine synthase, chloroplastic/chromoplastic, *O-*acetylserine sulfhydrylase in supplied leaves that turn into an up regulation of *O*-acetyl-*L*-serine, glutamate and glutamine. *O*-acetyl-*L*-serine is a direct precursor of cysteine biosynthesis and is hence crucial for sulfur assimilation, while glutamate and glutamine are involved in the biosynthetic pathway of glutathione (Hirai et al., 2003). Change in the oxidation state of Cys residues and its thiol group promotes the response to change in redox environments and enable an organism to adapt to stress conditions (Montrichard et al., 2009). Plants grown with 100 μM of MgSO_4_ started to up regulate the synthesis of cysteine and several free amino acids, as detected by gene expression (high level Glutamate/leucine/phenylalanine/valine dehydrogenases and Flavonol sulfotransferase-like) and Volcano analysis, and to activate the protein synthesis pathway (Durenkamp et al., 2007). Furthermore, the accumulation of both glutamate and glutamine suggests an increase in activity of the GS-GOGAT cycle, likely to support nitrogen assimilation and protein synthesis. Similar results were also found studying the response to sulfur stress in *Brassica napus* (Zhang et al., 2015).

## Conclusion

Our approach was able to identify and highlight the main determinants of sugar beet response to changes in sulfate availability. The combination of ionomics, morphological, metabolomics and molecular approaches appeared to be a particularly valuable system for the evaluation of sugar beet nutritional status under different nutrient availability conditions.

## Author Contributions

PS, CB, and GB wrote the main manuscript and performed the OA analysis. VM and GM developed the panel of TaqMan assays for the OA analysis. MC and MB conducted the ionomic analysis. LL performed the metabolomic analysis. VB, DP, and AB conducted the morphological analysis. GC, AS, and SN coordinated data collection and reviewed the manuscript.

## Conflict of Interest Statement

The authors declare that the research was conducted in the absence of any commercial or financial relationships that could be construed as a potential conflict of interest.

## Footnotes

1 pmn.plantcyc.org
